# Voluntary distance running prevents TNF-mediated liver injury in mice through alterations of the intrahepatic immune milieu

**DOI:** 10.1038/cddis.2017.266

**Published:** 2017-06-22

**Authors:** Yvonne Huber, Nadine Gehrke, Jana Biedenbach, Susanne Helmig, Perikles Simon, Beate K Straub, Ina Bergheim, Tobias Huber, Detlef Schuppan, Peter R Galle, Marcus A Wörns, Marcus Schuchmann, Jörn M Schattenberg

**Affiliations:** 1 I. Department of Medicine, University Medical Center of the Johannes Gutenberg University, Mainz, Germany; 2 Department of Sports Medicine, Rehabilitation and Prevention, Johannes Gutenberg University, Mainz, Germany; 3 Institute of Pathology, University Medical Center of the Johannes Gutenberg University, Mainz, Germany; 4 Department of Nutritional Sciences, Molecular Nutritional Science, University of Vienna, Vienna A-1090, Austria; 5 Department of General, Visceral and Transplant Surgery, University Medical Center of the Johannes Gutenberg University, Mainz, Germany; 6 Institute of Translational Immunology, University Medical Center of the Johannes Gutenberg University, Mainz, Germany; 7 Department of Medicine, Medical Center Konstanz, Konstanz, Germany

## Abstract

Physical activity confers a broad spectrum of health benefits. Beyond the obvious role in metabolically driven diseases, the role of physical activity in acute liver injury is poorly explored. To study the role of physical activity in acute liver injury, a novel model of voluntary distance running in mice was developed and mice were subjected to acute liver injury induced by *N*-galactosamine (GalN) and lipopolysaccharide (LPS). Analyses included histological stains, immunoblotting, qRT-PCR and FACS analysis. Voluntary distance running increased to an average of 10.3 km/day after a learning curve. Running lead to a decrease in the absolute numbers of intrahepatic CD4+ T and B lymphocytes and macrophages after 7 weeks. In parallel, hepatic mRNA expression of inflammatory cytokines including IL-6 and IL-1beta, TGF-beta and monocyte chemoattractant protein-1 (MCP-1/CCL2) were suppressed, while TNF-α was not affected by exercise. Likewise, expression of the macrophage-specific antigen F4/80 was downregulated 1.6-fold from exercise. Notably, acute liver injury from GaIN/LPS was significantly blunted following 7 weeks of voluntary exercise as determined by liver histology, a 84.6% reduction of alanine aminotransferase (*P*<0.01) and a 54.6% reduction of aspartate aminotransferase (*P*<0.05) compared with sedentary mice. Additionally, proinflammatory cytokines, activation of caspase 3 and JNK were significantly lower, while antiapoptotic protein A20 increased. Voluntary distance running alters the intrahepatic immune phenotype producing an environment that is less susceptible to acute liver injury.

Physical activity is known to have multiple beneficial health effects. Several studies have demonstrated health-promoting properties and a marked reduction in the risk of developing chronic disease including coronary artery disease, type 2 diabetes, stroke and cancer.^1, 2^ In line with these findings, individuals with a high degree of physical activity exhibit a decreased overall mortality.^3^ On the contrary, physical inactivity is a major cause of premature mortality through promotion of the metabolic syndrome.^4^ The mechanisms underlying the beneficial effects of exercise have been attributed to metabolic alterations, including the regulation of growth hormones, antioxidant defence pathways or alterations of systemic and organ-specific inflammation.^5^ In this line, serum CRP levels – indicative of chronic inflammation – within the US-based Health and Nutrition Examination Survey (NHANES 1999–2002), were inversely related to cardiorespiratory fitness.^6^

While the effects on general health are well explored, the impact of regular physical activity on the liver is less understood. Improvement of antioxidant- or anti-inflammatory capacities is in principal beneficial for a variety of acute or chronic liver diseases.^5, 7^ Few data implicate an influence of exercise on the release of anti-inflammatory cytokines from the musculature or visceral adipose tissue, and alterations of cortisol and adrenalin signaling as extrahepatic modulators in liver disease.^8^ Improvement of liver mitochondrial dysfunction which contributes to the generation of toxic lipids and reactive oxygen species could be involved in this protective mechanism.^9^ The strongest evidence for beneficial effects of physical activity in the liver has been generated in the context of non-alcoholic fatty liver disease and non-alcoholic steatohepatitis (NASH). Here regular exercise leads to an improvement of peripheral and hepatic insulin sensitivity, reduction of oxidative stress, decreased hepatic lipid content mediated in part through AMP-activated protein kinase (AMPK) activation and finally decreased inflammation and fibrogenesis.^10^ Specifically, recent clinical studies have convincingly demonstrated that a lifestyle intervention involving exercise and caloric restriction decreases body weight and insulin resistance and improves liver histology.^11^ Even in the advanced disease stage of compensated cirrhosis physical exercise was able to decrease the hepatic venous pressure gradient – a main predictor of mortality in these patients – likely due to decreasing hepatic inflammation and vascular stiffness.^12^ Animal models support these clinical findings. Voluntary wheel running was shown to decrease inflammation in adipose tissue and decrease the severity of NASH and hepatic fibrosis in both a genetic foz/foz^13^ and a dietary high-fat model^14^ of NASH.

Acute liver failure (ALF) is a rare but life-threatening illness with a high mortality, which can result from a variety of hepatic insults, characterized by loss of liver function and hepatic inflammation.^15^ In animal models, treatment with lipopolysaccharide (LPS) and the hepatocyte-specific transcriptional inhibitor *N*-acetyl-d-galactosamine (GalN) is used to mimic ALF. LPS activates hepatic resident tissue macrophages in the liver (Kupffer cells), which release inflammatory cytokines, especially tumor necrosis factor (TNF)-alpha, in the presence of GalN.^16^ TNF is a pleiotropic cytokine and its downstream actions are mainly mediated through the transcription factor nuclear factor kappa-B (NF-*κ*B). Deletion of NF-*κ*B or its regulatory subunits or its inhibition through GalN leads to acute liver injury from TNF-mediated caspase activation and from hepatocellular apoptosis.^17^ Mechanistic studies in transgenic animals have also shown a central involvement of pro- and antiapoptotic factors that regulate caspase activation.^18, 19^ In addition, mitogen-activated protein kinases (MAPK) and among these specifically c-Jun N-terminal kinase (JNK) are critically involved in cell death signaling in hepatocytes during ALF.^20^ An antiapoptotic factor that inhibits TNF-mediated apoptosis in hepatocytes is the protein A20.^21^ The aim of the current study was to examine the effects of voluntary exercise in C57BL/6 mice on key pro- and antiapoptotic factors and specifically inflammatory cells in the liver, and to investigate its role in influencing liver injury and ALF from TNF.

## Results

### Establishment of a model of voluntary distance running in mice

**Figure 1:**
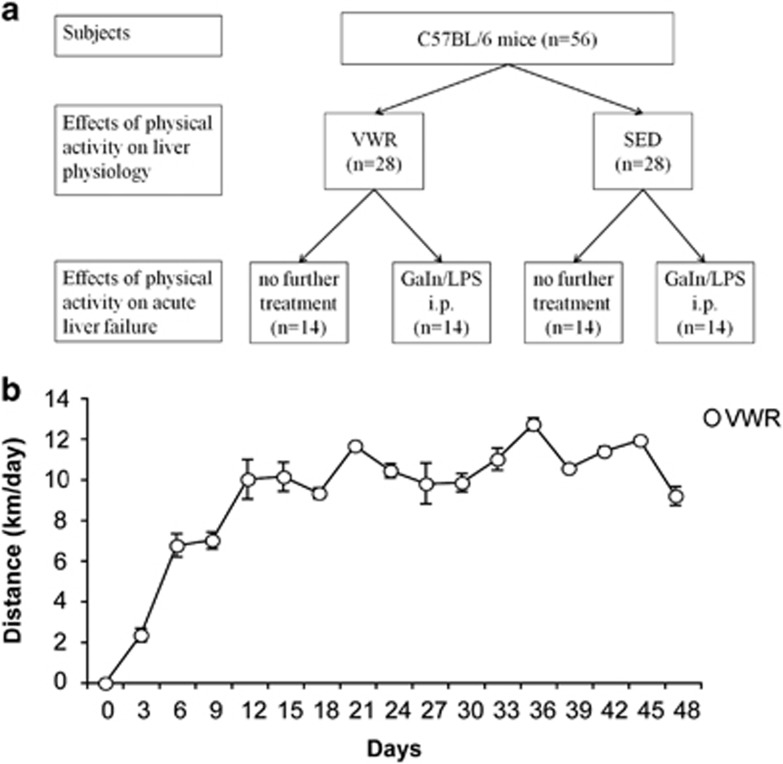
(**a**) Experimental setup. Male C57BL/6 mice (*n*=56), aged 7–11 weeks, were randomly assigned to a voluntary wheel running (VWR, *n*=28) group or sedentary (SED, *n*=28) group. After a mean of 45 days 14 mice of each group were killed to examine the effects of voluntary physical activity. The remaining 14 mice of each group were injected intraperitoneally (i.p.) with LPS and GaIN to examine the effects of physical activity on acute liver failure. (**b**) Running distance. VWR distance was monitored continuously using a bicycle tachometer in single housed mice. Median distance is displayed over time from *n*=28 VWR mice

Voluntary running in mice is variable and influenced by environmental factors.^22^ In order to assess the effects of running on liver pathophysiology, we examined mice following an exercise period after a median of 45 days and in a second experiment studied the effect of exercise on acute liver failure (Figure 1a). The average daily running distance in the voluntary wheel running (VWR) group was 10.3 km (range 3.75–15.73 km/day). Weekly running distance increased during the first 2 weeks and plateaued at 10.69 km (±0.38) from week 3 on (Figure 1b). During the experiments, weight gain was more pronounced in the sedentary (SED) group (VWR: +6.95%, *P*<0.01 *versus* SED: +10.01%, *P*<0.001; Table 1).

### Effects of VWR on hepatic metabolism and intrahepatic immune cell populations

To assess the effects of VWR in mice, the metabolic and immunological phenotype was examined. Serum cholesterol levels (before 115.4 mg/dl (±21.3) *versus* after 97.1 mg/dl (±17.7), *P*<0.001) and serum triglyceride levels (before 108.3 mg/dl (±34.5) *versus* after 58.0 mg/dl (±23.7), *P*<0.001) decreased significantly with exercise. Blood glucose levels and transaminases were unaffected by running, while cellular-free (cf) DNA decreased significantly after exercise. Table 1 summarizes the observed effects of voluntary distance running.

**Figure 2:**
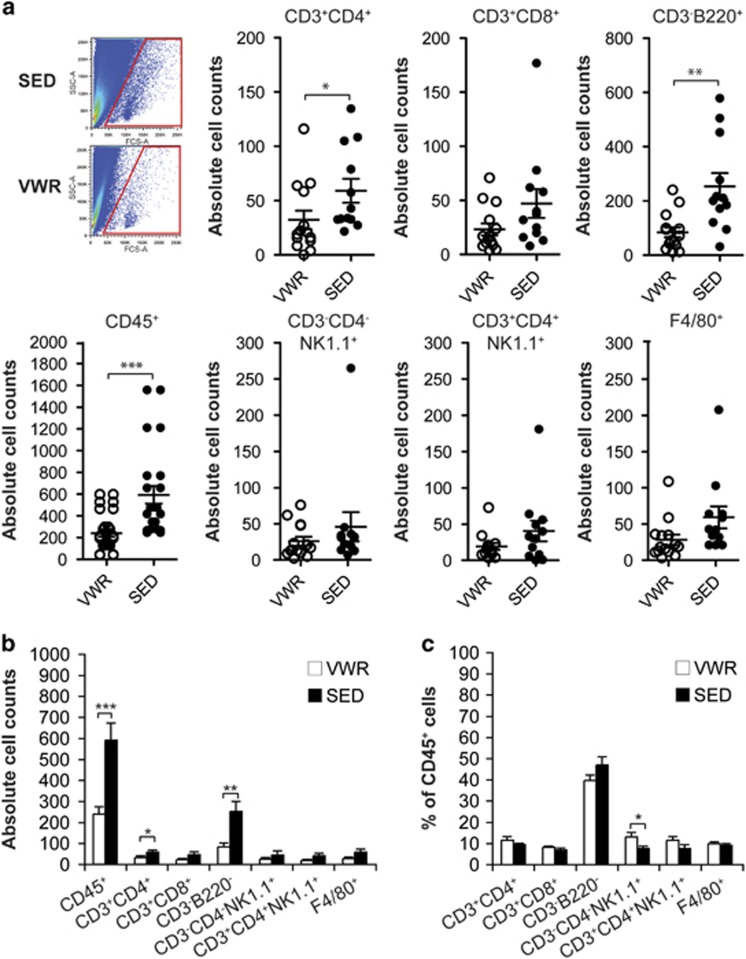
Effect of exercise on intrahepatic immune cells. (**a**) Living intrahepatic CD45+ cells were quantified by FACS, *n*=14 mice in voluntary wheel running (VWR) and *n*=12 mice in the sedentary (SED) group. A representative forward (FSC) and side scatter (SSC) dot plot of cells isolated from whole liver tissue of a single VWR and SED mouse is shown. Absolute (**a**and **b**) and relative counts (**c**) of intrahepatic leukocyte subsets were quantitated by gating on CD45+CD3+CD4+ or CD45+CD3+CD8+ for T cells, CD45+CD3− NK1.1−CD45R/B220+ for B cells, CD45+CD3−CD4−NK1.1+ for NK cells, CD45+CD3+CD4+NK1.1+ for NKT cells and CD45+CD4−CD8−F4/80+ for macrophages. In (**a**) data points depict individual animals and in (**b**) and (**c**) bars indicate mean±S.E.M.

Interestingly, voluntary running induced a robust effect on the intrahepatic immune phenotype of mice. In the VWR group a significant reduction of the absolute number of intrahepatic CD45+ leukocytes was observed by flow cytometric analysis (*P*<0.001; Figures 2a and b). Quantification of the different CD45+ leukocyte subpopulations revealed a global decrease in the absolute number of hepatic CD4+ and CD8+ T cells, B cells, NK cells, NKT cells and macrophages in the VWR group, whereby CD4+ T cell (*P*<0.05), B cell (*P*<0.01) and macrophage counts (*P*=0.08) were mostly prominently affected by running (Figures 2a and b). These data are in line with previous studies that showed mobilization of innate and adaptive immune cells from peripheral tissues into the circulation during exercise in rats.^23^ However, the relative proportions of different CD45+ leukocyte subpopulations remained unchanged with the exception of hepatic NK cells, whose ratio was significantly increased in the VWR compared with the SED group (*P*<0.05) (Figure 2c and Supplementary Figure S1). In parallel to the alteration of the intrahepatic immune profile, exercise led to changes in the expression of inflammatory markers in liver tissue. Mice in the VWR group showed decreased hepatic mRNA expression of inflammatory cytokines including IL-6 (3.17-fold, *P*<0.05), IL-1*β* (2.43-fold, NS), TGF-β (1.34-fold, NS) and MCP-1 (1.81-fold, *P*<0.01), whereas TNF mRNA was not altered by exercise. In line with these qRT-PCR data, the mRNA expression of the macrophage-specific antigen F4/80, which is encoded by *Adgre1*, was also suppressed by exercise (1.67-fold, NS) (Table 2, Supplementary Figure S2). In contrast to the intrahepatic cytokine milieu, no clear difference of systemic markers of inflammation including IL-6, IL-12p70, TNF, IFN-*γ* and MCP-1 were detectable in the serum between the SED and the VWR groups (Supplementary Table S1). Thus, voluntary running in mice exerts anti-inflammatory activity in the liver that potentially affects innate and adaptive immune effectors mechanisms.

Regulators of cellular metabolism and inflammation were examined in the hepatic tissue including AMPK – a regulator of glucose uptake and *β*-oxidation of fatty acids – acetyl-CoA carboxylase (ACC), the fatty acid synthase (FAS) and the transcription factor sterol regulatory element binding protein (SREBP)-1c. There was no difference between VWR and SED mice regarding transcript levels of ACC, AMPK and FAS, while exercise decreased the expression of SREBP-1c (*P*<0.05) (Supplementary Table S2).

### Physical activity prevents acute liver injury from GalN/LPS

**Figure 3:**
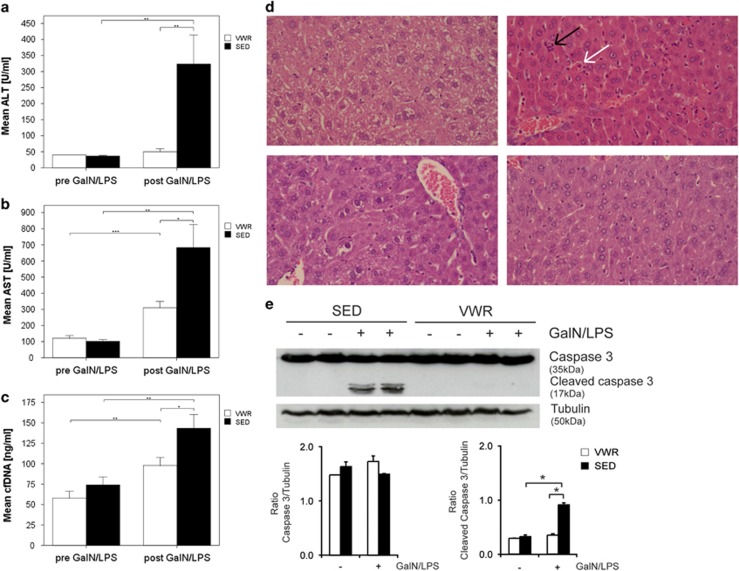
Physical activity prevents acute liver injury from GalN/LPS. (**a**) Alanine aminotransferase (ALT), (**b**) aspartate aminotransferase (AST) and (**c**) cellular-free DNA (cfDNA) measured in mice after voluntary wheel running (VWR; *n*=14) and in the sedentary group (SED; *n*=14) and 5 h after i.p. injection with GalN/LPS (each group VWR and SED *n*=14). Data are means±S.E.M. (**d**) Representative hematoxylin and eosin-stained liver sections from mice in the sedentary (SED) and voluntary wheel running (VWR) group following treatment with or without GaIN/LPS (magnification each × 400). Inflammatory cell aggregates (black arrow) and necrotic hepatocytes (white arrow) are denoted. (**e**) Activation of caspase 3 in the voluntary wheel running (VWR) and sedentary (SED) groups following GaIN/LPS treatment. A representative blot and densitometric analysis are provided

To determine the functional effects of the observed immune alterations, a model of acute liver injury was employed in sedentary and active mice. GaIN/LPS was administered at a fixed dose intraperitoneally and resulted in a 96.9-fold increase of endotoxin levels at 5 h irrespective of the level of activity (Supplementary Table S3). Following GaIN/LPS treatment serum alanine aminotransferase (ALT) increased 8.8-fold and aspartate aminotransferase (AST) levels increased 6.7-fold in the SED group (Figures 3a and b), whereas transaminases in the VWR group were significantly lower. Thus VWR caused an 84.6% reduction of ALT (*P*<0.01) and 54.6% reduction of AST (*P*<0.05) compared with the SED group. Impairment of hepatic function, resulting in hypoglycemic, was detectable in all animals regardless of the activity level. Nonetheless, the decrease in fasting blood glucose was less severe in the VWR compared with the SED group (VWR *versus* SED: 101.1 mg/dl (±22.7) *versus* 82.4 mg/dl (±21.4), *P*<0.05).

Histological analysis showed severe liver injury with central grouped necrosis, hemorrhage, inflammatory cell infiltrates and apoptotic hepatocytes following GalN/LPS treatment. These histological alterations were less pronounced in mice in the VWR group (Figure 3d and Supplementary Table S4). Liver injury from GalN/LPS was characterized by activation of caspases. In parallel to the reduction of transaminases and histological changes, activation of caspase 3 was significantly blunted in mice in the VWR group following GaIN/LPS treatment (Figure 3e). Importantly, there were no significant pre- and post-intervention differences in weight between the two groups (Table 1).

### GalN/LPS-induced markers of inflammation and chemotaxis are significantly reduced by voluntary running

**Figure 4:**
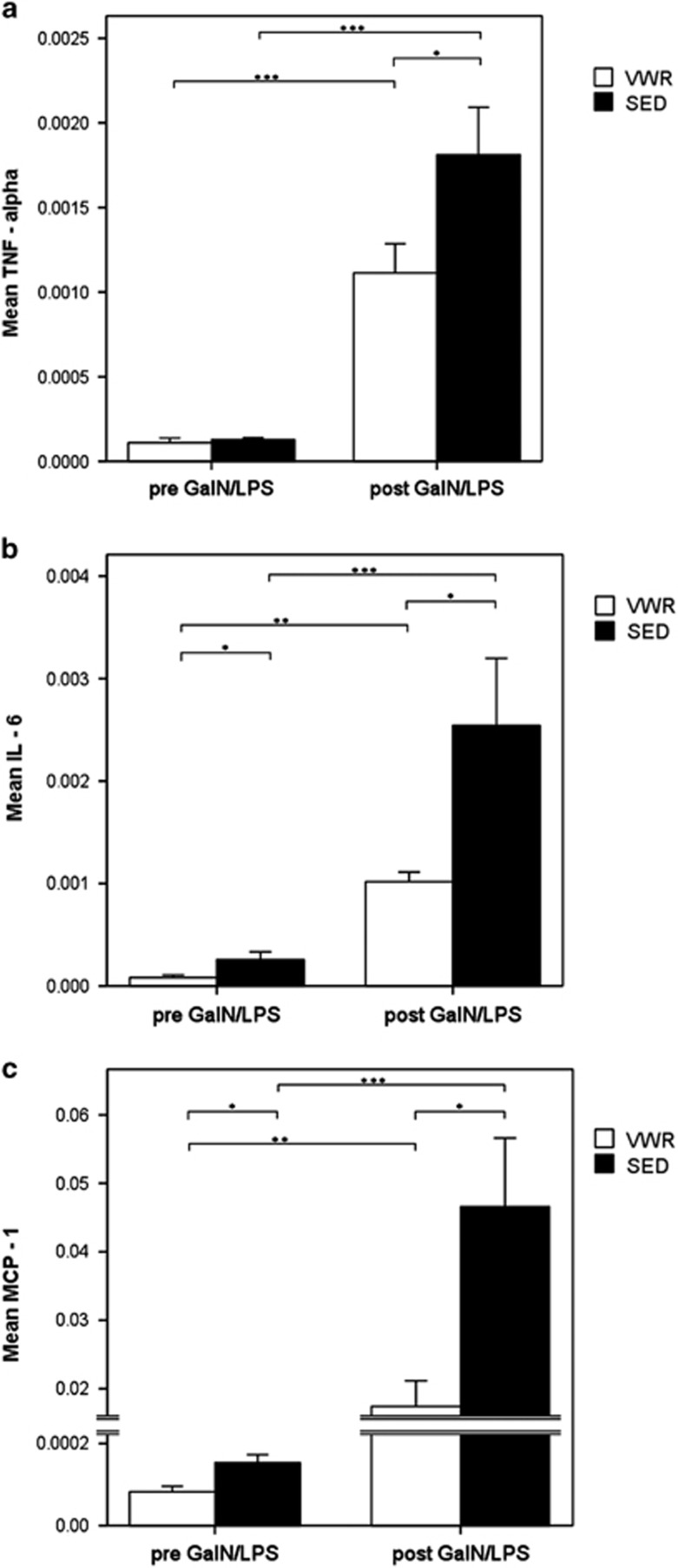
Reduced hepatic expression of inflammatory cytokine and chemokine from exercise. Relative hepatic mRNA expression of (**a**) TNF, (**b**) IL-6 and (**c**) MCP-1 at baseline and 5 h after GalN/LPS injection in VWR and SED mice. Data are means of *n*=9 in every group±S.E.M.

The release of inflammatory cytokines and chemokines is responsible for liver injury observed from GalN/LPS. Following injection the hepatic expression levels of TNF, IL-6 and MCP-1 increased significantly. However, VWR mice exhibited a significantly reduced hepatic expression of proinflammatory and chemotactic cytokines following GalN/LPS compared with the SED group: TNF by 44.4% (*P*<0.05), IL-6 by 60.0% (*P*<0.05) and MCP-1 by 63.8% (*P*<0.05) (Figures 4a–c). Additionally, serum concentrations of TNF, IL-6 and MCP-1 following GalN/LPS challenge were examined. Although GalN/LPS induced a sharp increase in serum cytokines, exercise did not significantly influence these serum levels (Supplementary Table S1). Thus, the anti-inflammatory effects of exercise in this model of acute liver failure are restricted to the hepatic compartment.

### Hepatic JNK activation is blunted by physical activity in GalN/LPS-induced liver injury

**Figure 5:**
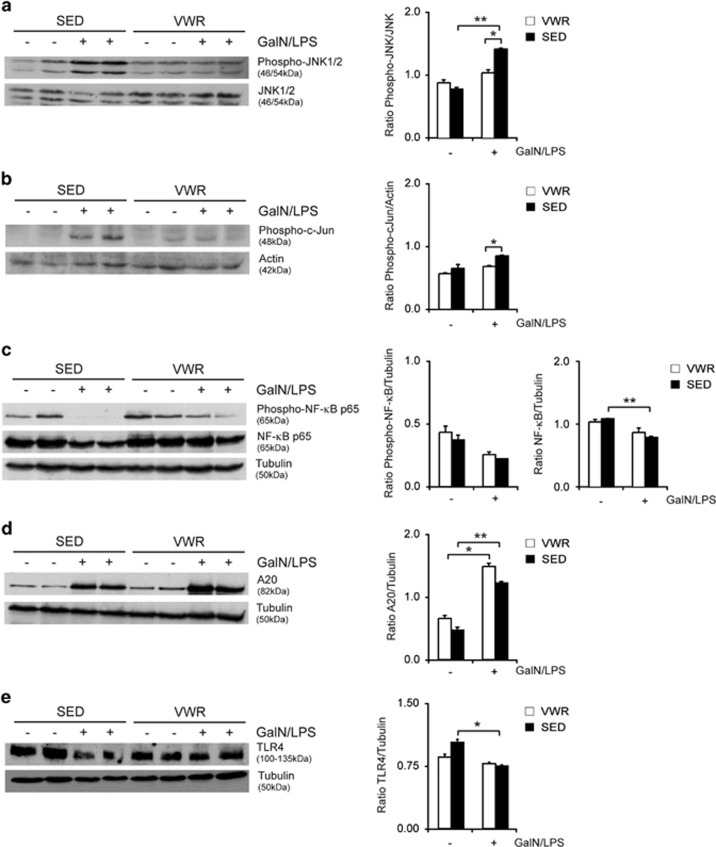
Protection from acute liver failure following exercise involves MAP kinases and NF-κB signaling as well as downregulation of TLR4 protein expression. Liver tissue of unchallenged and GalN/LPS-challenged mice in the voluntary wheel running (VWR) and sedentary group (SED) were harvested after 5 h and levels of (**a**) phosphorylated and total JNK, (**b**) phosphorylated c-Jun, (**c**) total and phosphorylated NF*κ*B p65, (**d**) A20 and (**e**) TLR4 were determined by immunoblotting (representative western blots are shown)

To assess the molecular mechanisms of the beneficial effects of physical activity in acute liver injury, stress kinase signaling pathways were examined. The MAPK JNK has been shown to regulate apoptotic liver injury.^20, 24^ More recently, inhibition of apoptosis signal-regulating kinase 1 (ASK1) using selonsertib, an upstream kinase that regulates JNK and p38,^25^ has been implicated as a treatment of chronic liver disease. GaIN/LPS treatment resulted in increased phosphorylation of p54 and p46 isoforms and its downstream effector c-Jun, while levels of total protein were unaffected. JNK and c-Jun phosphorylation from GalN/LPS were both decreased in the VWR compared with the SED group (Figures 5a and b), reflecting decreased activation of this pro-injurious signaling pathway. JNK signaling is controlled through the transcription factor NF-*κ*B, a central regulator of inflammation, liver cell injury and proliferation.^26^ Previous studies have repeatedly shown that NF-*κ*B is a major negative regulator of programmed cell death, especially in response to TNF and blockade of NF-*κ*B activation promotes TNF-induced cell death.^27^ GalN/LPS decreased NF-*κ*B p65 phosphorylation. This effect was blunted by exercise in the VWR group (Figure 5c).

A primary mechanism through which NF-*κ*B suppresses JNK signaling involves the select activation of antiapoptotic proteins, including the zinc-finger protein A20. NF-*κ*B upregulates expression of A20, which acts upstream of MAPK to reduce the degree of liver injury by suppressing, for example, JNK activation.^28^ Voluntary exercise lead to an increase of the antiapoptotic protein A20 expression, which was even augmented following GaIN/LPS treatment and significantly higher in mice in the VWR compared with the SED group (Figure 5d).

### Effects of physical activity on damage-associated molecular patterns

Activation of the innate immune system during cellular injury and cell death results from the release of endogenous damage-associated molecular patterns (DAMPs) such as heat-shock proteins (HSPs), high mobility group box-1 (HMGB1) and cell free (cf) DNA, from activated, stressed or dead cells. These are detected by pattern recognition receptors (PRRs) including, among others, cell surface and intracellular toll-like receptors (TLR) as well as cytosolic nucleic acid receptors to promote the inflammatory response. We have previously shown an involvement of TLR9 and stimulator of interferon genes (STING) in triggering acute, apoptotic liver injury by recognizing DAMPs.^29^ To determine the involvement of DAMPs during exercise and liver injury, cfDNA was measured. In the VWR group cfDNA decreased slightly after exercise (before 103.3 ng/ml (±51.3) *versus* after 72.5 ng/ml (±32.9), *P*<0.05), an effect that was not observed in the SED group (before 102.5 ng/ml (±55.6) *versus* after 80.3 ng/ml (±42.7), *P*=0.12; Table 1). Treatment with GaIN/LPS resulted in increased levels of cfDNA in both groups, while the increase in the SED group was significantly higher compared with exercising mice (VWR *versus* SED: 97.8 ng/ml (±36.6) *versus* 143.4 ng/ml (±62.4), *P*<0.05) (Figure 3c).

Hepatic gene expression of *Tlr9* and *Tmem173* – encoding the endosomal and cytosolic DNA sensors TLR9 and STING – were downregulated following liver injury. While this was expected following application of GalN, the effect was more pronounced in the SED group. Likewise, downregulation of TLR4 gene expression was observed following liver injury, with a more significant decrease in SED mice (Table 3). Consistently, western blot analysis confirmed a reduction of TLR4 protein following GalN/LPS in the liver from SED mice that was less prominent in mice following exercise (Figure 5e).

## Discussion

The current study assessed the liver-specific effects of voluntary exercise in mice and its influence on acute, inflammatory liver failure. While the beneficial effects of physical exercise are well established and lifestyle changes are the basic recommendation for all diseases that emerge in the context of metabolic risk factors,^1^ the influence of exercise on the liver has not been addressed to the same extent. Here we show that endurance exercise altered the intrahepatic immune phenotype resulting in a reduction in the absolute number of CD45+ leukocytes. This included all leukocyte subtypes and produced a significant reduction in the number of CD4+ T cells, B cells and a shift in the relative immune cell numbers, with increasing NK cells. This phenotype was accompanied by reduced expression of inflammatory cytokines including IL-6, IL-1beta, TGF-beta and MCP-1 in the not injured liver. Interestingly, these changes were not detectable in the serum to the same extent in the absence of liver injury. This is different from the findings in obese individuals when expanding visceral adipose tissue (VAT) stores and NASH promote a chronic low-grade state of inflammation with elevated serum levels of TNF, IL-1, IL-6 and other cytokines in the peripheral blood.^30^ Although several studies have explored the relationship of physical activity and the immune system in recent years, no data regarding the hepatic phenotype have been available.

Thus the predominantly studied compartments include the VAT and skeletal muscle. In these, an anti-inflammatory phenotype characterized by a shift towards alternatively activated (M2-polarized) macrophages and the presence of CD4+ regulatory T cells is promoted by exercise. Physical inactivity on the other hand promotes the infiltration of an expanding VAT with proinflammatory M1-polarized macrophages and T cells and stimulates the release of inflammatory cytokines.^31^ The sympathetic nervous system is a second important regulator of immunity and inflammation and the release of cortisol and adrenaline from the adrenal gland block the release of inflammatory cytokines, for example, TNF, while IL-6, produced by contracting skeletal muscle, is able to counter regulate TNF secretion.^32^ IL-6 also induces the release of IL-1 receptor antagonist (IL-1RA) from monocytes and macrophages, thus increasing the circulating concentrations of this anti-inflammatory cytokine antagonist. In leptin-deficient ob/ob mice, norepinephrine regulated intrahepatic NKT cells and protected from LPS-induced liver injury.^33^ Finally, exercise regulated and suppressed the expression of proinflammatory TLR4 in monocytes.^31^ These effects occurred independently of weight loss and promoted an improvement of tissue hypoxia, and reduced expression of leukocyte adhesion and cytokine production in endothelial cells.^34^

The exercise models reported in the literature vary with regard to the intensity and distance of running and can be influenced by individual and environmental factors, for example, age and stress.^13, 35, 36^ Increasing leukocytes and NK cell counts have been reported in the blood of athletes during exercise,^37, 38^ while a decrease in the numbers of peripheral immune cells – predominately CD4 T cells and NK cells – has been observed during the recovery phase of exercise.^39^ Overall, our observations in exercising *versus* sedentary mice are well in line with these rodent and human data.

Notably, the change of the intrahepatic immune phenotype protected mice from ALF as demonstrated by reduced amounts of hepatic cell death, reduced activation of caspase 3 and a reduction of ALT and AST. Also hypoglycemic in response to GalN/LPS treatment – a sign for impairment of hepatic function – was significantly less pronounced in active mice. Inflammatory cytokines were significantly increased following GalN/LPS treatment while the absolute expression levels of cytokines in exercising mice were significantly lower compared with sedentary animals. These results are in line with published data on the role of exercise in decreasing hepatic IL-6 signaling and mRNA expression of other inflammatory markers including TNF, MCP-1, IL-10 and IL-1*β* in LPS-induced inflammation.^36^ The current study therefore expands previous observations of the beneficial effect of exercise on LPS-induced shock and liver injury, but goes beyond to identify the intrahepatic mechanisms and signaling pathways that are linked to the observed protection. We showed that physical activity prevented NF-*κ*B inactivation and blocked prolonged JNK activation in hepatocytes. NF-*κ*B activation is critical for the protection of hepatocytes against TNF and loss NF-*κ*B function results in spontaneous, TNF-mediated inflammation and hepatocellular injury.^40^ Likewise prolonged JNK activation promotes apoptotic cell death in hepatocytes.^41, 42^ In the current study the antiapoptotic protein A20 was an important NF-*κ*B regulated effector controlled by exercise. Forced expression of A20 was previously shown to prevent TNF-induced prolonged JNK activation and cell death in several models.^43, 44^ In our study we were also able to identify decreased release of cfDNA – a cell death-associated molecular pattern (cDAMP) – in the context of ALF following physical exercise. cDAMPs are recognized among others through TLR9 and STING and promote the secretion of proinflammatory cytokines and chemokines. Treatment with GaIN/LPS caused the release of cfDNA. Interestingly, in the VWR group this release of small, uniform DNA fragments was diminished, likely due to lower levels of liver injury. These molecules exhibit proinflammatory activity and are capable to amplifying liver cell death and inflammation. In line with reduced cfDNA levels in exercised mice we also observed lower levels of PRRs including TLR9, STING and TLR4, which act as sensors of PAMPs and DAMPs during liver injury. These data support the notion that exercise influences the expression of immune receptors in the liver.

We chose an endurance type of exercise and a washout phase of 24 h prior to analysis in male mice aged 7–11 weeks. The average distance covered per day was 10.25 km following an adaptation period of 3 weeks. Within the exercise group, both under- and over-performers were identified; however, individual distance did not correlate with the degree of protection from acute liver injury, arguing against a threshold but rather demonstrating a beneficial effect of a personalized aerobic, endurance training. Muscle mass is a likely critical factor that contributes to the observed anti-inflammatory effects, for example, through the release of anti-inflammatory IL-6.^10^ In the current study, weight gain was comparable in both the sedentary and the active groups over time. Although no data on body composition are available, it can be assumed that increasing weight was partly explained by an increase in muscle mass in the exercise group. These findings are backed by the clinical observation that sarcopenia is associated with advanced liver disease and that inflammatory NASH appears to be more pronounced with sarcopenia.^45, 46^ Also, lipid profiles were affected more strongly in the exercise group. A decrease of triglycerides and cholesterol was observed over time and was more pronounced in mice that were exercising.

The functional effects of the immune shift are likely to impact health even beyond acute liver failure. Thus, a lower incidence of colon cancer from physical activity has been observed in epidemiological studies.^47^ In mice an increase of NK cells and NK cell activity from voluntary running was shown to contribute to the suppression of DEN-induced hepatocellular carcinoma (HCC).^48^ Likewise, in a transgenic model of HCC involving the deletion of PTEN physical activity decreased the number, size and proliferation rate of HCC independently of steatohepatitis.^49^

In summary, physical activity altered the intrahepatic immune and cytokine milieu to an extent that resulted in the protection from acute, inflammatory liver injury. The current study delineates novel, liver-specific effects of physical exercise and its role in regulating anti-inflammatory pathways including shifts in intrahepatic immune cells and reduced MAPK kinase activation. These results encourage exercise in patients with chronic liver disease and support the importance of the intrahepatic immune phenotype in response to liver injury.

## Materials and methods

### Animals

Animals were bred at the animal facility of the University Medical Center Mainz, according to the criteria outlined by the 'Guide for the Care and Use of Laboratory Animals'. The study was conducted following approval by the Landesuntersuchungsamt Rheinland-Pfalz. Male C57BL/6 mice, aged 7–11 weeks, were randomly assigned to a voluntary wheel running (VWR) group or a sedentary (SED) group. There was no difference in age between both groups: mean age of the SED animals 8.25±1.6 weeks and of the VWR animals 8.5±1.3 weeks (*P*=0.52). The VWR mice (*n*=28) were individually housed in cages (size 43 cm in length × 25 cm width and 28 cm height) and outfitted with a 11.5 cm diameter running wheel. Wheel running activity was continuously recorded using a usual bicycle tachometer (Ciclosport). Sedentary mice were housed individually in smaller cages (34  l × 22 w × 13 h cm) in the absence of environmental enrichment. All mice were kept on a 12-h light/dark cycle with free access to food and water for the duration of the study. Mice were fed 'complete feed for mouse breeding' (Mouse breeding, Extrudate V1126-000; ssniff Spezialdiäten GmbH, Soest, Germany). Body weight was measured weekly. After a mean of 45 days 14 mice of each group were killed to examine the effects of voluntary physical activity. This included serological testing, immunohistochemistry, immunoblotting, qRT-PCR and FACS analyses. The remaining 14 mice of each group were injected intraperitoneally (i.p.) with LPS and GaIN. The running wheel was removed 24 h prior to the initiation of liver injury (washperiod) to avoid confounding effects of physical activity.^50^ Mice received a fix dose of 10 *μ*g LPS (from '*Escherichia coli* Serotype 026:B6, L-8274;' Sigma-Aldrich, Steinheim, Germany) and 5 mg galactosamine (GaIN, from 'd-(+)-galactosamine hydrochloride G1639' Sigma-Aldrich, Steinheim, Germany) i.p. and were killed for further analysis at 5 h as previously published.^18^ The experimental setup is shown in Figure 1a.

### Serological analysis

Blood was collected from anesthetized mice by retrobulbar venous plexus puncture using glass micro-hematocrit tubes at the indicated time points during the experiment and by cardiac puncture from anesthetized mice at the end of the study. Serum ALT, AST, lactate dehydrogenase, cholesterol levels and triglycerides levels were measured using a standard clinical analyzer (Hitachi 917; Roche, Mannheim, Germany).

### Histological analyses

A middle section of the right liver lobe was preserved for histological evaluation following hematoxylin and eosin staining using standard protocols. Histological sections were evaluated blinded by an experienced histopathologist (BS). Representative images were taken using an Olympus BX45 microscope (Hamburg, Germany) with a Jenoptik PROGRES GRYPHAX camera (Micro Optimal, Kirchheim/Teck, Germany). Histological scoring were performed in high-power fields (HPFs) and scored the following items. Inflammation: low: minimal portal inflammation with 3–5 single-cell necrosis/15 HPF and no group necrosis; medium: medium portal inflammation with 6–9 single-cell necrosis/15 HPF and/or maximum one group necrosis; high: pronounced portal inflammation with more than 10 single-cell necrosis/15 HPF and/or more than one group necrosis.

### Quantitative real-time PCR

Isolation of total RNA, cDNA synthesis and qRT-PCR were performed as previously described.^18^ Roche LightCycler software (LightCycler 480 Software Release 1.5.0) was used to perform advanced analysis relative quantification using the 2(−ΔΔC(T)) method. Expression data were normalized to the reference gene *Gapdh* (primers from Qiagen, Hilden, Germany). Primer sequences (all Eurofins Genomics, Ebersberg, Germany) are listed in Supplementary Table S5.

### Immunoblotting and immunohistochemistry

Primary antibodies included A20, caspase 3, cleaved caspase 3, phospho-c-Jun, JNK, phospho-JNK, NF-*κ*B, phospho-NF-κB, TLR4 (all Cell Signaling Technology Inc., Danvers, MA, USA), actin (Santa Cruz Biotechnology, Santa Cruz, CA, USA) and alpha-tubulin (Sigma-Aldrich, Steinheim, Germany). Membranes were exposed to anti-mouse, anti-goat (both DAKO Denmark A/S, Glostrup, Denmark) or anti-rabbit (Santa Cruz Biotechnology) secondary antibodies conjugated with horseradish peroxidase. Densitometric analyses derived from three blots are included in addition to a representative immunoblot.

### Quantitative analysis of cytokines and chemokines

Concentrations of serum cytokines and chemokines were measured by cytometric bead array Mouse Inflammation Kit (BD Biosciences, Heidelberg, Germany) using a BD FACS Canto II flow cytometer (BD Biosciences). Analysis was performed by FCAP Array v3 Analysis software (Soft Flow, St. Louis Park, MN, USA).

### FACS analysis of intrahepatic immune cells

Intrahepatic leukocytes were isolated and subjected to flow cytometric analysis (FACS) as previously described.^51^ All antibodies were purchased from BioLegend (San Diego, CA, USA).

### DNA extraction, quantification and cfDNA analysis

Fifty microliters of plasma were diluted with 250 *μ*l phosphate-buffered saline (Life Technologies, Darmstadt, Germany) to a total volume of 300 *μ*l. About 1/100 vol of Triton X-100 (Carl Roth, Karlsruhe, Germany) were added, samples were incubated at 98 °C for 5 min and then cooled on ice for 5 min. Samples were mixed with 1 vol phenol : chloroform : isoamyl alcohol, pH 8.0 (Sigma-Aldrich, Taufkirchen, Germany), vortexed for 30 s and centrifuged at 20 °C, 16 000 × *g* for 10 min. The upper aqueous phase was pipetted off and DNA was precipitated with 2.5 vol of 100% ethanol, 1/10 vol 3 M sodium acetate, pH 5.2 and 20 *μ*g Glycogen (Life Technologies GmbH, Darmstadt, Germany) overnight at −20 °C. The next day, the precipitate mixture was centrifuged at 4 °C, 16 000 × *g* for 30 min. DNA pellets were washed two times with 70% ethanol and a third time with 100% ethanol. After each washing step the samples were centrifuged at 4 °C, 16 000 × *g* for 5 min. Pellets were dried for about 20 min at 55 °C and eluted with 20 *μ*l TE buffer, pH 8.0 (Life Technologies GmbH). Samples were further incubated at 37 °C for 30 min to completely dissolve the DNA. Measurements were performed using the NanoDrop 3300 (Thermo Fisher Scientific Inc., Waltham, MA, USA). The specimen (5 *μ*l) was mixed with PicoGreen (5 *μ*l) and incubated for 4 min in the absence of light. The solution was transferred to NanoDrop in 2 *μ*l portions, resulting in five measurements of each specimen. Quantification was performed by comparison to a standard curve which was previously created using Lambda-DNA (Invitrogen, Life Technologies GmbH) as defined by the manufacturer.

### LPS measurement

To measure endotoxin levels, K3EDTA plasma or serum samples were diluted with endotoxin-free water and heated at 70 °C for 20 min. Plasma levels of endotoxin were determined using a commercially available limulus amebocyte lysate assay with a concentration range of 0.015–1.2EU/ml (Charles River, L’Arbaesle, France) in the lab of IB as detailed before.^52^

### Statistical analysis

Values are given as mean±standard error of the mean (S.E.M.) and represent data from a minimum of three independent experiments. The *F*-test was used to verify the assumption of equal variances, and two-tailed Student’s *t*-test was used to determine statistical significance. Statistically significant values are presented as *P<0.05, **P<0.01, *** *P*<0.001.

## Figures and Tables

**Table 1:** Effects of voluntary wheel running on body weight, liver function tests and metabolism

	**VWR (*n*=28)**			**SED (*n*=28)**			**VWR *versus* SED**
	**Pre- intervention**	**Post-intervention**	***P***	**Pre- intervention**	**Post-intervention**	***P***	***P* post-intervention**
Weight (g)	25.5 (±2.1)	27.3 (±1.9)	**<0.01**	24.8 (±2.8)	27.3 (±2.0)	**<0.001**	0.93
Cholesterol (mg/dl)	115.4 (±21.3)	97.1 (±17.7)	**<0.001**	118.0 (±20.9)	105.5 (±25.9)	0.05	0.44
Triglyceride (mg/dl)	108.3 (±34.5)	58.0 (±23.7)	**<0.001**	94.7 (±37.3)	74.4 (±37.5)	**<0.05**	0.06
Glucose (mg/dl)	138.2 (±43.1)	149.3 (±58.8)	0.44	141.7 (±36.5)	143.1 (±52.1)	0.92	0.68
ALT (U/ml)	36.4 (±7.8)	40.6 (±3.0)	**0.01**	36.4 (±7.8)	38.3 (±6.3)	0.33	0.09
AST (U/ml)	117.4 (±65.6)	145.4 (±91.5)	0.19	113.1 (±56.2)	122.0 (±63.4)	0.58	0.27
cfDNA (ng/ml)	103.3 (±51.3)	72.5 (±32.9)	**0.02**	102.5 (±55.6)	80.3 (±42.7)	0.12	0.47

**Table 2:** Hepatic expression of inflammatory cytokines

	**VWR (*n*=9)**	**SED (*n*=9)**	**VWR** ***versus*** **SED *P***
TNF	1.1E−04 (±8.1E−05)	1.3E−04 (±3.5E−05)	0.56
IL-6	8.2E−05 (±7.6E−05)	2.6E−04 (±2.3E−04)	**<0.05**
IL-1b	3.5E−03 (±9.4E−04)	8.5E−03 (±2.8E−03)	0.18
TGF-b	4.1E−03 (±6.3E−04)	5.5E−03 (±6.0E−04)	0.18
MCP-1	8.3E−05 (±4.0E−05)	1.5E−04 (±5.8E−05)	**<0.01**
F4/80	1.5E−02 (±5.4E−03)	2.5E−02 (±1.9E−03)	0.17

**Table 3:** Effects of voluntary wheel running on hepatic expression levels of cytokines, NF*κ*B and pattern recognition receptors in TNF-induced liver injury

	**VWR**			**SED**			**Post-GaIN/LPS VWR** ***versus*** **SED**
	**Pre- GaIN/LPS**	**Post-GaIN/LPS**	***P***	**Pre- GaIN/LPS**	**Post-GaIN/LPS**	***P***	***P***
TNF	1.1E−04 (±8.1E−05)	1.1E−03 (±5.4E−04)	**<0.001**	1.3E−04 (±3.5E−05)	1.8E−03 (±8.9E−04)	**<0.001**	**<0.05**
IL-6	8.2E−05 (±7.6E−05)	1.0E−03 (±2.9E−04)	**<0.001**	2.6E−04 (±2.3E−04)	2.5E−03 (±2.1E−03)	**<0.01**	**<0.05**
MCP-1	8.3E−05 (±4.0E−05)	1.7E−02 (±1.2E−02)	**<0.01**	1.5E−04 (±5.8E−05)	4.7E−02 (±3.2E−02)	**<0.01**	**<0.05**
NF*κ*B	1.9E−03 (±7.8E−04)	1.2E−02 (±3.7E−03)	**<0.01**	2.9E−02 (±2.2E−03)	1.3E−02 (±3.9E−03)	**<0.001**	0.6
TLR4	4.6E−03 (±5.9E−03)	6.5E−04 (±3.6E−04)	0.05	1.1E−02 (±1.3E−02)	7.9E−04 (±4.2E−04)	**<0.05**	0.45
TLR9	2.5E−03 (±4.2E−03)	2.4E−04 (±1.8E−04)	0.13	5.4E−03 (±6.7E−03)	3.4E−04 (±2.9E−04)	**<0.05**	0.35
STING	5.2E−03 (±8.0E−03)	1.4E−03 (±4.1E−04)	0.2	8.0E−03 (±1.0E−02)	2.1E−03 (±7.2E−04)	0.09	**<0.05**

For TNF, IL-6 and MCP-1 *n*=9 mice were used in each group, for NF*κ*B, TLR4, TLR9 and STING *n*=11 mice were used in each group.
