# Supplementary figures and images for: Voluntary distance running prevents TNF-mediated liver injury in mice through alterations of the intrahepatic immune milieu
Source: Cell Death Dis. 2017 Jun 22;8(6):e2893–. doi: 10.1038/cddis.2017.266 (PMC5520921; doi:10.1038/cddis.2017.266)

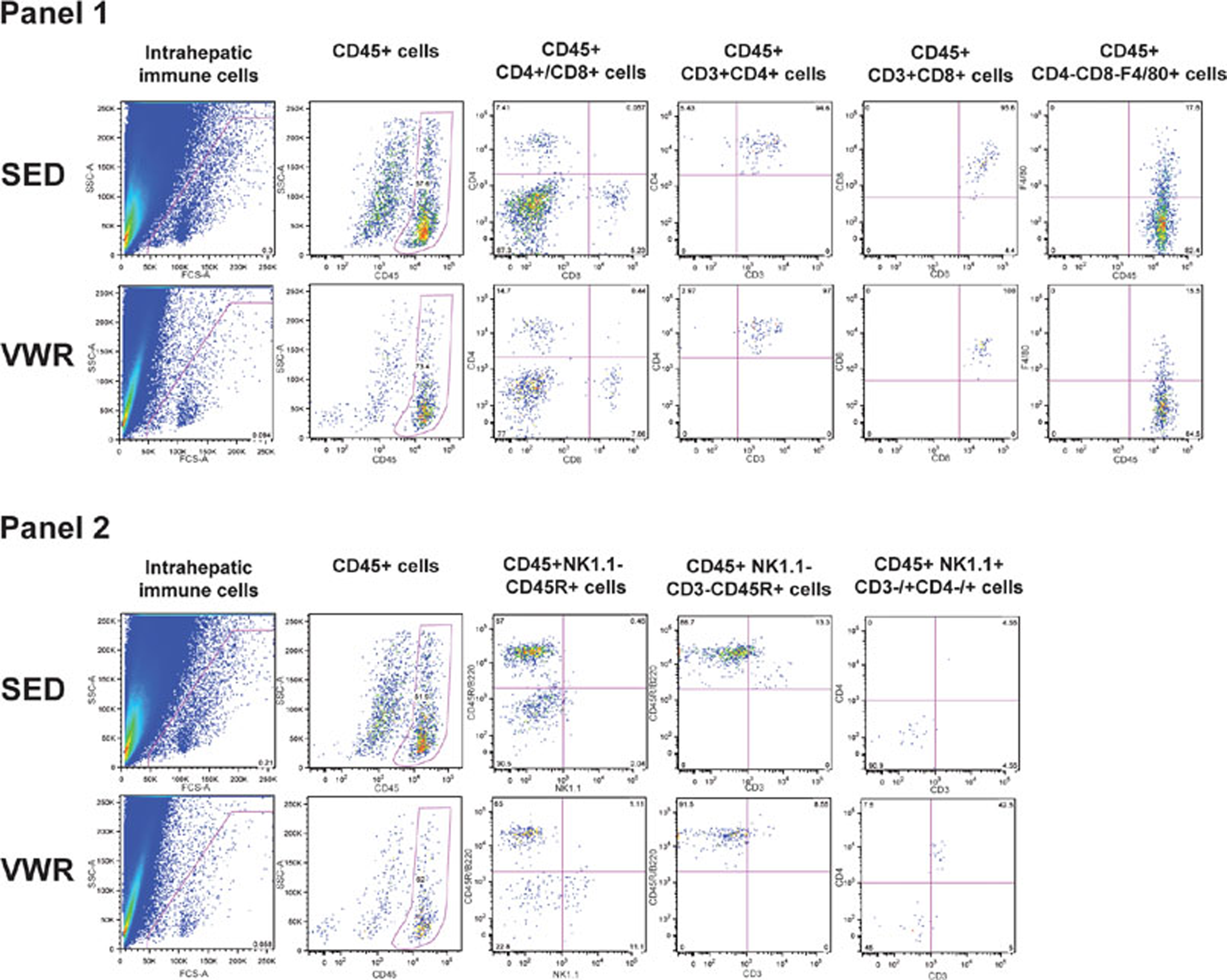

Supplement: Supplementary Figure 1 [file cddis2017266x1.tif]
