# Supplementary material for: Voluntary distance running prevents TNF-mediated liver injury in mice through alterations of the intrahepatic immune milieu
Source: Cell Death Dis. 2017 Jun 22;8(6):e2893–. doi: 10.1038/cddis.2017.266 (PMC5520921; doi:10.1038/cddis.2017.266)
Supplement: Supplementary Figure 2 [file cddis2017266x2.docx]

**Supplementary Figure 2: Hepatic expression of inflammatory cytokines for TNF, IL-6 and MCP-1 in individual mice.** Each point represents the expression of the depicted cytokine relative to GAPDH expression in this specific mouse.
