# Supplementary material for: Voluntary distance running prevents TNF-mediated liver injury in mice through alterations of the intrahepatic immune milieu
Source: Cell Death Dis. 2017 Jun 22;8(6):e2893–. doi: 10.1038/cddis.2017.266 (PMC5520921; doi:10.1038/cddis.2017.266)
Supplement: Supplementary Table 1 [file cddis2017266x3.docx]

**Suppl. Table 1:** Systemic markers of inflammation in mice with (VWR) or without (SED) running and after liver injury with GalN/LPS were measured by cytometric bead array (CBA).

|  | VWR | | | SED | | | pre-  GaIN/LPS  VWR vs. SED | post-  GaIN/LPS  VWR vs. SED |
| --- | --- | --- | --- | --- | --- | --- | --- | --- |
|  | **pre- GaIN/LPS** | **post-**  **GaIN/LPS** | **p** | **pre- GaIN/LPS** | **post-**  **GaIN/LPS** | **p** | **p** | **p** |
| TNF | 6.88 (±9.73) | 199.55 (±49.21) | **<0.001** | 2.11 (±2.67) | 174.39 (±136.23) | 0.08 | 0.57 | 0.74 |
| IL-6 | 176.05 (±126.64) | 6897.73 (±2575.00) | **<0.01** | 39.67 (±49.77) | 4732.70 (±3152.27) | **<0.05** | 0.29 | 0.27 |
| MCP-1 | 199.48 (±203.56) | 1923.52 (±355.72) | **<0.01** | 59.46 (±40.04) | 1867.90 (±410.76) | **<0.01** | 0.44 | 0.83 |
| IFN-γ | 0 | 47.37 (±58.75) | **-** | 0 | 96.42 (±163.42) | **-** | - | 0.51 |
| IL-10 | 0 | 103.78 (±48.51) | - | 0 | 114.16 (±128.56) | **-** | - | 0.86 |
| IL-12p70 | 3.35 (±4.74) | 32.10 (±18.23) | **<0.05** | 21.80 (±30.83) | 10.46 (±3.39) | 0.69 | 0.49 | **0.03** |

For pre-GaIN/LPS n=2 mice were used in each group; for post GaIN/LPS n=4 mice were used in the SED group and n=6 mice in the VWR group.
