# Supplementary material for: Voluntary distance running prevents TNF-mediated liver injury in mice through alterations of the intrahepatic immune milieu
Source: Cell Death Dis. 2017 Jun 22;8(6):e2893–. doi: 10.1038/cddis.2017.266 (PMC5520921; doi:10.1038/cddis.2017.266)
Supplement: Supplementary Table 2 [file cddis2017266x4.docx]

**Supplement Table 2:** Alterations of major regulators of cellular metabolism and inflammation from exercise.

|  | VWR (n=14) | SED (n=14) | VWR vs. SED  p-value |
| --- | --- | --- | --- |
| AMPK | 1.2E-02 (±1.2E-02) | 2.2E-02 (±2.9E-02) | 0.23 |
| ACC | 4.27E-03 (±1.09E-03) | 3.65E-03 (±3.94E-04) | 0.14 |
| FAS | 6.37E-02 (±1.3E-01) | 8.19E-02 (±2.1E-01) | 0.79 |
| SREBP-1c | 4.6E-03 (±2.04E-03) | 7.79E-03 (±3.5E-03) | **0.03** |

The absolute expression of AMP-activated protein kinase (AMPK), acetyl-CoA carboxylase (ACC), fatty acid synthase (FAS) and sterol regulatory element binding protein (SREBP)-1c mRNA levels normalized to GAPDH in each individual mouse is displayed in sedentary (SED) and exercising (voluntary wheel running; VWR).
