# Supplementary material for: Voluntary distance running prevents TNF-mediated liver injury in mice through alterations of the intrahepatic immune milieu
Source: Cell Death Dis. 2017 Jun 22;8(6):e2893–. doi: 10.1038/cddis.2017.266 (PMC5520921; doi:10.1038/cddis.2017.266)
Supplement: Supplementary Table 3 [file cddis2017266x5.docx]

**Supplementary Table 3**: Levels of endotoxin in TNF-induced acute liver injury

|  |  |  |
| --- | --- | --- |
| **Mouse** | **Group** | **Level of endotoxin**  **(EU/ml)** |
|  |  |  |
|  |  |  |
| 1 | SED, untreated | 0.13 |
| 2 | SED, treated | 12.82 |
| 3 | SED, treated | 10.30 |
| 4 | SED, treated | 12.91 |
| 5 | SED, treated | 14.11 |
| 6 | VWR, treated | 12.97 |
| 7 | VWR, treated | 13.08 |
| 8 | VWR, treated | 12.96 |
| 9 | VWR, treated | 11.61 |
|  |  |  |

Levels of endotoxin was measured in a subgroup of 9 mice 5 hours after intraperitoneally (i.p.) injection with 10µg lipopolysaccharide (LPS) and 5mg galactosamine (GaIN); SED: sedentary group, VWR: voluntary wheel running group.
