# Supplementary material for: Voluntary distance running prevents TNF-mediated liver injury in mice through alterations of the intrahepatic immune milieu
Source: Cell Death Dis. 2017 Jun 22;8(6):e2893–. doi: 10.1038/cddis.2017.266 (PMC5520921; doi:10.1038/cddis.2017.266)
Supplement: Supplementary Table 4 [file cddis2017266x6.docx]

**Suppl. Table 4**: Histological characteristics in sedentary and exercising mice and following GalN/LPS-induced liver injury.

| **Mouse number** | **± GaIN/LPS** | **Inflammation** | **Necrosis** | **evaluation** |
| --- | --- | --- | --- | --- |
| 1 | SED, untreated | none | none | normal, minimal steatosis |
| 2 | SED, untreated | none | none | normal, minimal steatosis |
| 3 | SED, untreated | none | none | normal, minimal steatosis |
| 4 | VWR, untreated | medium | >10/HPF single-cell and 1-2/HPF group necrosis | numerous single-cell and group necrosis, no steatosis, no portal inflammation |
| 5 | VWR, untreated | none | none | low steatosis |
| 6 | VWR, untreated | none | none | low steatosis |
| 7 | SED, treated | high | >10/HPF single-cell and 3-4/HPF group necrosis | numerous single-cell and group necrosis, about 35% of parenchymal areas, no steatosis, high portal inflammation |
| 8 | SED, treated | high | >10/HPF single-cell and 2-3/HPF group necrosis | numerous single-cell and group necrosis, about 20% of parenchymal areas, no steatosis, high portal inflammation |
| 9 | SED, treated | low | few single-cell and group necrosis | few single-cell and group necrosis, no steatosis, low inflammation |
| 10 | SED, treated | high | >10/HPF single-cell and 1-2/HPF group necrosis | numerous single-cell and group necrosis, no steatosis, high portal inflammation |
| 11 | SED, treated | high | >10/HPF single-cell and 1-2/HPF group necrosis | numerous single-cell and group necrosis, no steatosis, high portal inflammation |
| 12 | SED, treated | low | few single-cell and group necrosis | few single-cell and group necrosis, no steatosis, low inflammation |
| 13 | VWR, treated | none | none | normal |
| 14 | VWR, treated | none | none | normal |
| 15 | VWR, treated | none | none | normal, minimal steatosis |
| 16 | VWR, treated | none | none | normal |
| 17 | VWR, treated | low | few single-cell necrosis | few single-cell necrosis, no steatosis, no inflammation |
| 18 | VWR, treated | high | >10/HPF single-cell and 4-5/HPF group necrosis | numerous single-cell and group necrosis, about 50% of parenchymal areas, no steatosis, high portal inflammation |

Histological analysis of mice in a sedentary (SED) and exercise (voluntary wheel running; VWR) group. A subgroup of mice was injected intraperitoneal with saline (untreated) or GaIN/LPS (treated) and examined following 4 hours. The detailed histological assessment is detailed in Material and Methods.
