# Supplementary material for: Voluntary distance running prevents TNF-mediated liver injury in mice through alterations of the intrahepatic immune milieu
Source: Cell Death Dis. 2017 Jun 22;8(6):e2893–. doi: 10.1038/cddis.2017.266 (PMC5520921; doi:10.1038/cddis.2017.266)
Supplement: Supplementary Table 5 [file cddis2017266x7.docx]

**Suppl. Table 5**: Primer sequences used in qRT-PCR analyses

| Gene | Protein | Forward primer | Reverse primer |
| --- | --- | --- | --- |
| *Adgre1* | F4/80 | CTT TGG CTA TGG GCT TCT AGT C | GCA AGG AGG ACA GAG TTT ATC GTG |
| *Acaca* | ACC | AAC ATC CCC ACG CTA AAC AG | CTG ACA AGG TGG CGT GAA G |
| *Ccl2* | CCL2 | CTT CTG GGC CTG CTG TTC A | CCA GCC TAC TCA TTG GGA TCA |
| *Fasn* | FAS | CCC TTG ATG AAG AGG GAT CA | GAA CAA GGC GTT AGG GTT GA |
| *Il1b* | IL-1β | TCT TTG AAG TTG ACG GAC CC | TGA GTG ATA CTG CCT GCC TG |
| *Il6* | IL-6 | AGT TGC CTT CTT GGG ACT GA | TTC TGC AAG TGC ATC ATC GT |
| *Prkaa1* | AMPK | TGT TCC AGC AGA TCC TTT CC | ATA ATT GGG TGA GCC ACA GC |
| *Srebf1* | SREBP-1c | ATC TCC TAG AGC GAG CGT TG | TAT TTA GCA ACT GCA GAT ATC CAA G |
| *Tgfb2* | TGF-β | TTC CTG GCG TTA CCT TGG T | CCA CTG CCG GAC AAC T |
| *Tmem173* | STING | CCT AGC CTC GCA CGA ACT TG | CGC ACA GCC TTC CAG TAG C |
| *Tlr4* | TLR4 | GCT TTC ACC TCT GCC TTC AC | GAA ACT GCC ATG TTT GAG CA |
| *Tlr9* | TLR9 | GAA AGC ATC AAC CAC ACC AA | ACA AGT CCA CAA AGC GAA GG |
| *Tnf* | TNF | GAA GTT CCC AAA TGG CCT CC | GTG AGG GTC TGG GCC ATA GA |
